# Reversal of Jaundice in Two Patients with Inoperable Cholangiocarcinoma Treated with Cisplatin and Gemcitabine Combination

**DOI:** 10.1155/2012/138381

**Published:** 2012-03-20

**Authors:** Maarten Criel, Filip Geurs, Siegfried Ponette, Katrien Bulte, Johan Ponette

**Affiliations:** ^1^Department of Medical Oncology, Regionaal Ziekenhuis SINT MARIA, Ziekenhuislaan 100, 1500 Halle, Belgium; ^2^Department of Gastroenterology, Regionaal Ziekenhuis SINT MARIA, Ziekenhuislaan 100, 1500 Halle, Belgium

## Abstract

Two patients are presented with severe jaundice, due to inoperable cholangiocarcinoma. The chemotherapeutic approach in patients with severe jaundice is discussed. Many schedules of chemotherapy were developed in this tumor type with normal serum bilirubin. We report here the first successful use of cisplatin and gemcitabine combination chemotherapy in these patients. Tolerability was good and liver function tests gradually improved.

## 1. Introduction

The increase in number of patients with cholangiocarcinoma poses specific problems for the diagnostic strategy as well as for an increasing need for specific therapy [1]. The aggressive nature as well as its localisation of the tumor causes its typical presentation with jaundice. Its lack of operative options requires often a chemotherapeutic approach [2]. However, little is known about the use of chemotherapy in jaundiced patients.

## 2. Case Reports

**Figure 1:**
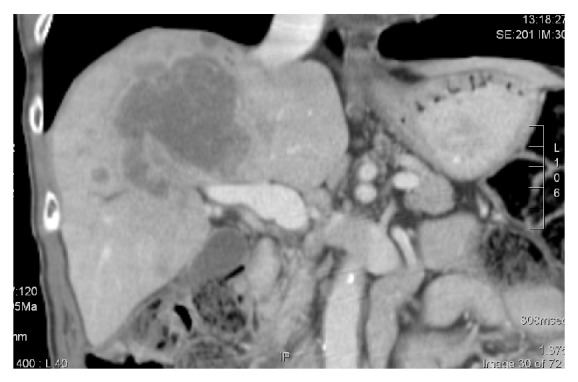
Initial CT scan in patient A, showing central cholangiocarcinoma with multiple satellite nodules surrounding the primary tumor.

**Figure 2:**
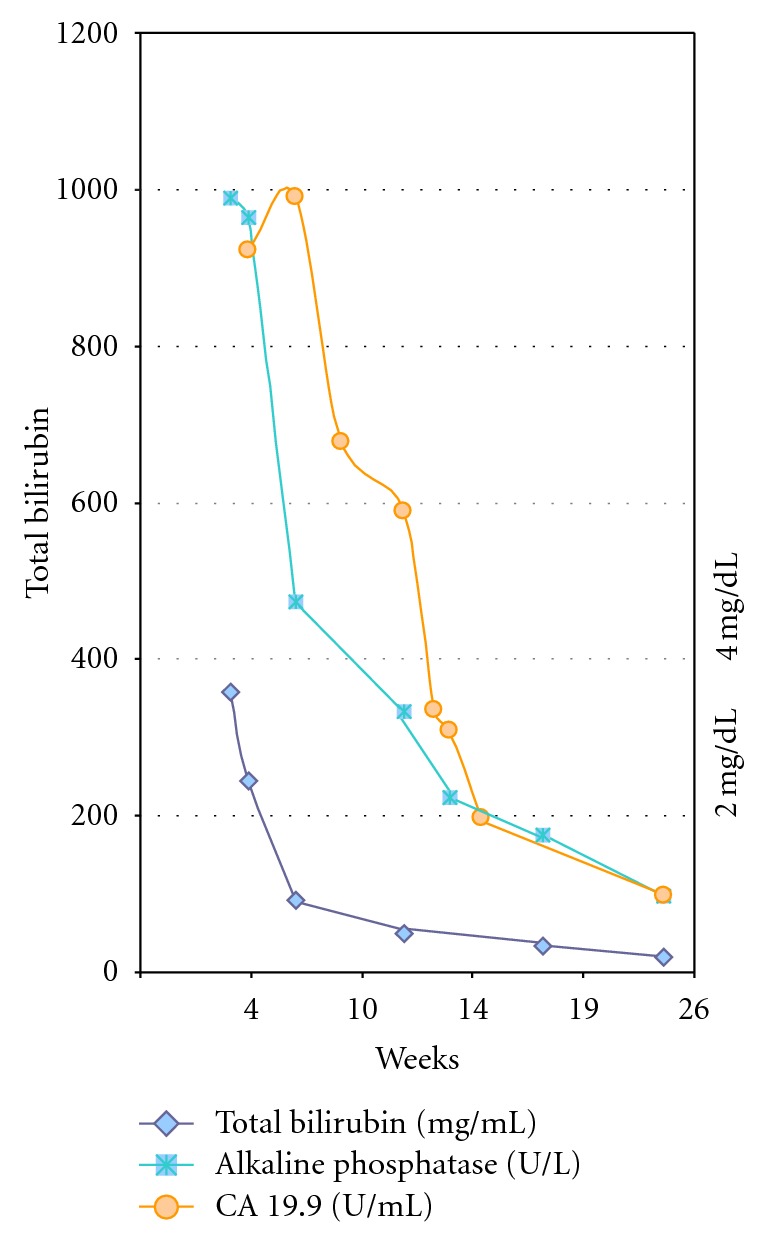
Evolution of liver function tests and tumor marker (CA 19.9) in patient A.

Patient A, 67-year-old Caucasian, presented with jaundice and was found at laparoscopy to have multiple intrahepatic metastases (Figure 1). Biopsy showed cholangiocarcinoma. Because of jaundice that could not be relieved surgically, the patient was transferred to the medical oncology ward. Because of the bilirubin level of 3.58 mg/dL at admission, we started with a lower dose of gemcitabine 800 mg/m² on day 1 and cisplatin 25 mg/m². Bilirubin level dropped at day 4 allowing for an increase of gemcitabine to 1 g/m² on day 8. Subsequent chemotherapies were done on an outpatient basis (gemcitabine 1 g/m² days 1 +8 and cisplatin 25 mg/m² days 2 +9 q 3w). Liver test all normalised (Figure 2) and CA 19.9 almost normalised. After 8 months of treatment, ascites developed due to progressive neoplastic disease, and patient subsequently received hospice care and died 10 months after diagnosis.

**Figure 3:**
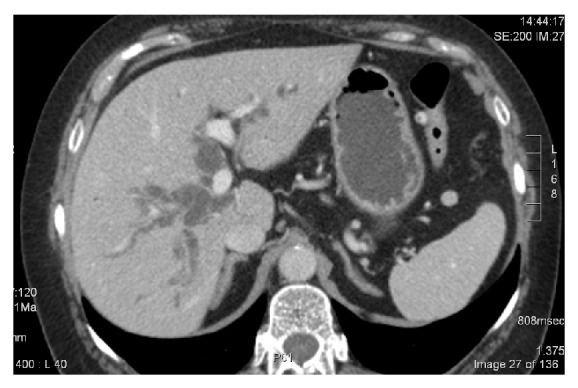
Impressive bile duct dilatation in patient B before stenting.

**Figure 4:**
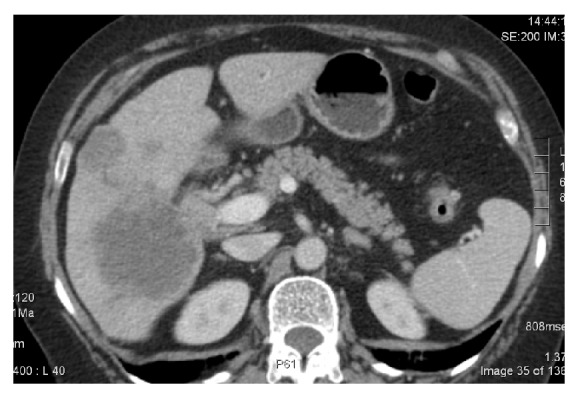
The underlying cholangiocarcinoma with intrahepatic metastasis and hilar extension in patient B.

**Figure 5:**
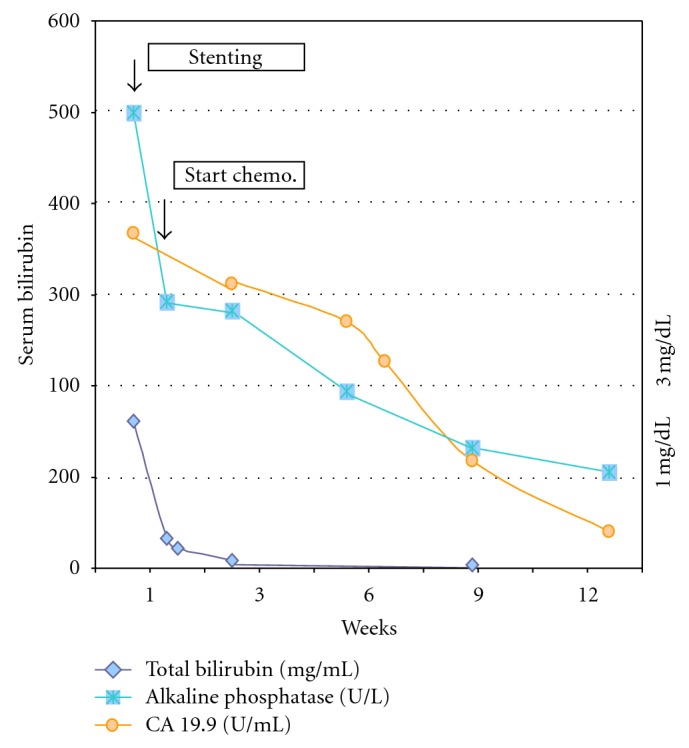
Evolution of liver function test and tumormarker (CA 19.9) in patient B.

**Figure 6:**
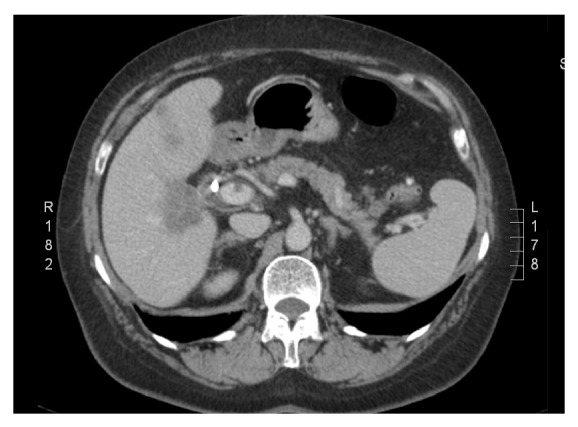
Partial response of the enormous mass in the right liver lobe.

Patient B is a 67-year-old housewife, who presents with progressive jaundice (bilirubin of 16 mg/dL) due to an underlying neoplasm of the bile duct (Klatskin type 1). CT scan showed intrahepatic metastases (Figures 3 and 4), and liver biopsy showed cholangiocarcinoma. After Ercp and stenting, bilirubin remained elevated at 3.2 mg/dL. Chemotherapy with gemcitabine-cisplatin (initially 800 mg/m² on day 1 and 1 g/m² on day 8, followed by cisplatin 25 mg/m² on days 2 +9) was administered without any significant toxicity. Liver function tests normalised, and CA 19.9 dropped ten fold (Figure 5). The control CT-scan after six months of treatment showed partial response; the patient was referred for liver surgery, and she is awaiting a right hepatectomy (Figure 6).

For localized cholangiocarcinoma, a multitude of treatments [3] were developed. Maximal surgery, included extensive resections and liver transplants, was developed in reference centers [4]. Local treatment comprising endoprosthesis in the biliary tract and local photodynamic treatment relieves jaundice and bile duct compression [5–7]. Also combined chemotherapy and radiotherapy are reported [8].

Chemotherapy represents the cornerstone of management for patients with inoperable cholangiocarcinoma [9]. The literature on chemotherapy in advanced cholangiocarcinoma is difficult to interpret because of the heterogeneity of cholangiocarcinoma, the use of various chemotherapeutic agents in different combinations and dosing regimens, and the small size of existing patient cohorts [10]. A combination of cisplatin and gemcitabine is, according to the most recent phase 3 trial [11], the only effective treatment at hand. Other smaller series report on the use of oxaliplatin, capecitabin, and 5 fluorouracil [12, 13]. All these combinations are, however, only published with normal heart and renal function and notably, bilirubin level below 2 mg/dL. Most patients, even after stenting, present with bilirubin levels over 2 mg/dL [14] as was the case in our second patient. This illustrates the lack of data in patients with end organ failure, notably liver failure and jaundice, like we previously reported [15].

 Although gemcitabine-cisplatin combination represents the most accepted chemotherapy regimen for biliary cancers at present, very few studies so far have looked at the feasibility and results in cholangiocarcinoma patients with jaundice. The initial doses of gemcitabine we used in both patients (800 mg/m²) represent a 20% reduction of the maximally tolerated dose of 1 g/m² [11] but retains significant effectiveness and is therefore also used in elderly patients with bladder cancer. The weekly use of cisplatin at 35 mg/m², two weeks out of three, is safe and effective as we have shown in another patient series with cisplatin monotherapy in severe jaundice [15].

## 3. Conclusion

We demonstrated in two patients with severe jaundice due to metastatic cholangiocarcinoma that cisplatin and gemcitabine combination chemotherapy can be safely and effectively administered. Both patients had similar response and tolerance as in patients with normal bilirubin.
